# Male pheromone protein components activate female vomeronasal neurons in the salamander *Plethodon shermani*

**DOI:** 10.1186/1471-2202-7-26

**Published:** 2006-03-22

**Authors:** Celeste R Wirsig-Wiechmann, Lynne D Houck, Jessica M Wood, Pamela W Feldhoff, Richard C Feldhoff

**Affiliations:** 1 Department of Cell Biology, University of Oklahoma Health Sciences Center, 940 S.L. Young Boulevard, Oklahoma City, OK 73104, USA; 2 Department of Zoology, Oregon State University, Corvallis, OR 97331-2914, USA; 3 Department of Biochemistry and Molecular Biology, University of Louisville Health Sciences Center, Louisville, KY 40292, USA

## Abstract

**Background:**

The mental gland pheromone of male Plethodon salamanders contains two main protein components: a 22 kDa protein named Plethodon Receptivity Factor (PRF) and a 7 kDa protein named Plethodon Modulating Factor (PMF), respectively. Each protein component individually has opposing effects on female courtship behavior, with PRF shortening and PMF lengthening courtship. In this study, we test the hypothesis that PRF or PMF individually activate vomeronasal neurons. The agmatine-uptake technique was used to visualize chemosensory neurons that were activated by each protein component individually.

**Results:**

Vomeronasal neurons exposed to agmatine in saline did not demonstrate significant labeling. However, a population of vomeronasal neurons was labeled following exposure to either PRF or PMF. When expressed as a percent of control level labeled cells, PRF labeled more neurons than did PMF. These percentages for PRF and PMF, added together, parallel the percentage of labeled vomeronasal neurons when females are exposed to the whole pheromone.

**Conclusion:**

This study suggests that two specific populations of female vomeronasal neurons are responsible for responding to each of the two components of the male pheromone mixture. These two neural populations, therefore, could express different receptors which, in turn, transmit different information to the brain, thus accounting for the different female behavior elicited by each pheromone component.

## Background

Chemosensory signals between conspecific animals, or pheromones, are important factors in orchestrating reproductive behaviors. A pheromone can be comprised of a single chemical [1], but more commonly is a mixture of chemicals in insects, amphibians and mammals [2-6]. In fact, certain pheromone components, such as frontalin or 1,5-dimethyl-6,8-dioxabicyclo [3.2.1]octane, are identical in species as diverse as the bark beetle, *Coleoptera: Scolytidae *[7] and the Asian elephant, *Elephas maximus *[6]. Frequently, mixtures of pheromonal components do not act optimally unless their components occur in exact proportions within the pheromonal mixture [8,9]. Also, individual components of pheromone mixtures frequently produce varying effects that differ from the effects of the mixture itself [10].

Frogs [11] and salamanders [12-14] use pheromones as attractants for mates during the mating season. Many salamanders also use pheromones during courtship, after potential mates already have been brought together [15]. A salamander model of pheromone influences on courtship behavior has been established [16-18]. We are using this model to study the neural pathways involved in pheromone communication. During the courtship behavior of the terrestrial salamander, *Plethodon shermani*, the male delivers a pheromone mixture from his mental gland to the female's snout. The pheromone increases the female's receptivity to the male as indicated by shorter mating times [16,18]. The pheromone mixture contains two main protein components, a 22 kDa protein called plethodon receptivity factor (PRF; [19]) and a 7 kDa protein now called plethodon modulatory factor (PMF; originally described as a ~10 kDa protein [19]). These proteins have been isolated and purified. They can be delivered individually to the female's snout during mating with a male whose mental gland has been surgically removed to test for individual pheromone effects. Behaviorally, the two proteins together [17] and PRF alone [18,19] facilitate the female's response to male courtship behavior by shortening the mating time. In contrast, PMF experimentally delivered alone lengthens the courtship time [20].

Pheromones delivered to the snout of the female are taken directly into the vomeronasal organ by the capillary action of a nasolabial groove. The vomeronasal organ transduces and transmits "large molecule" information to the brain in terrestrial vertebrates. This system has classically been thought to function in the transmission of pheromone information to the brain [21]. However, recent evidence suggests that the main olfactory system can also carry pheromonal signals to the brain in conjunction with the vomeronasal system, probably for the purpose of perception and localization [22,23]. The vomeronasal organ may have developed as a separate structure for the purpose of transporting compounds that could not normally be delivered to the olfactory system and/or that required a different neural route to specific brain nuclei involved in physiological responses to odors. While research has been conducted for some time now on insect and mammalian pheromones, recent work has begun to explore pheromones and their detection systems in other vertebrates such as amphibians [4,17].

In the current study, we tested the hypothesis that each of the protein components, PRF and PMF, of the male *P. shermani *pheromone activates vomeronasal neurons. In a previous study, neuronal uptake of agmatine was used as an indicator of vomeronasal responsiveness to the whole pheromone mixture [24]. Agmatine is a modified amino acid that can pass through nonspecific cation channels during neural activation [25]. Since PRF and PMF have opposite effects on behavior, the possibility exists that each compound may act differently in the vomeronasal organ. In this study we found that PRF and PMF each activate populations of vomeronasal receptor neurons.

## Results

### *Experiment 1. PRF application *– Histological observations

**Figure 1:**
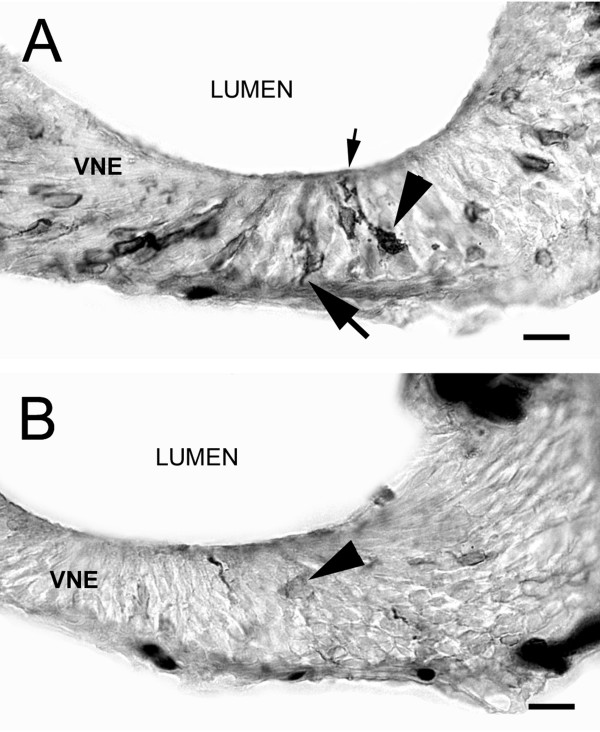
**Micrographs of immunocytochemically labeled coronal sections of female *P. shermani *vomeronasal epithelium (VNE) demonstrating agmatine uptake by stimulation with PRF (A) or a saline control solution (B). **(A) Application of PRF resulted in the labeling of numerous vomeronasal receptor neurons (arrow head illustrates one of the labeled cell bodies) throughout all depths of the epithelial layer. Labeling extended from the dendritic knob at the epithelial surface (small arrow) to the proximal portion of the axon (large arrow) within the organ. PRF application resulted in moderate-to-heavy intensity of labeling. (B) Application of saline produced very few labeled vomeronasal receptor neurons (arrow head) and these were very lightly labeled. Bars = 40 μm.

**Figure 2:**
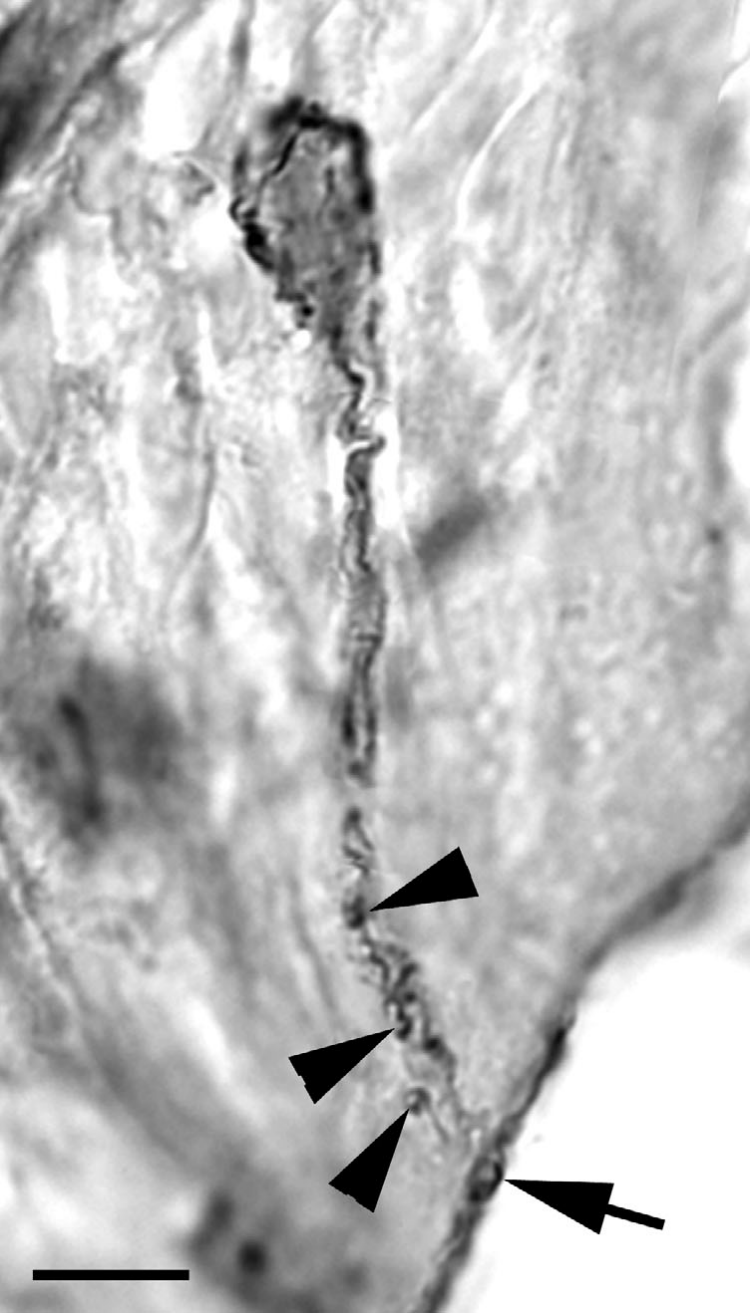
**High magnification micrograph of an agmatine-labeled vomeronasal neuron stimulated with PRF**. Arrowheads indicate areas of small vesicular aggregates of label. Arrow points to the dendritic knob on the surface of the epithelium. Bar = 10 μm.

**Figure 3:**
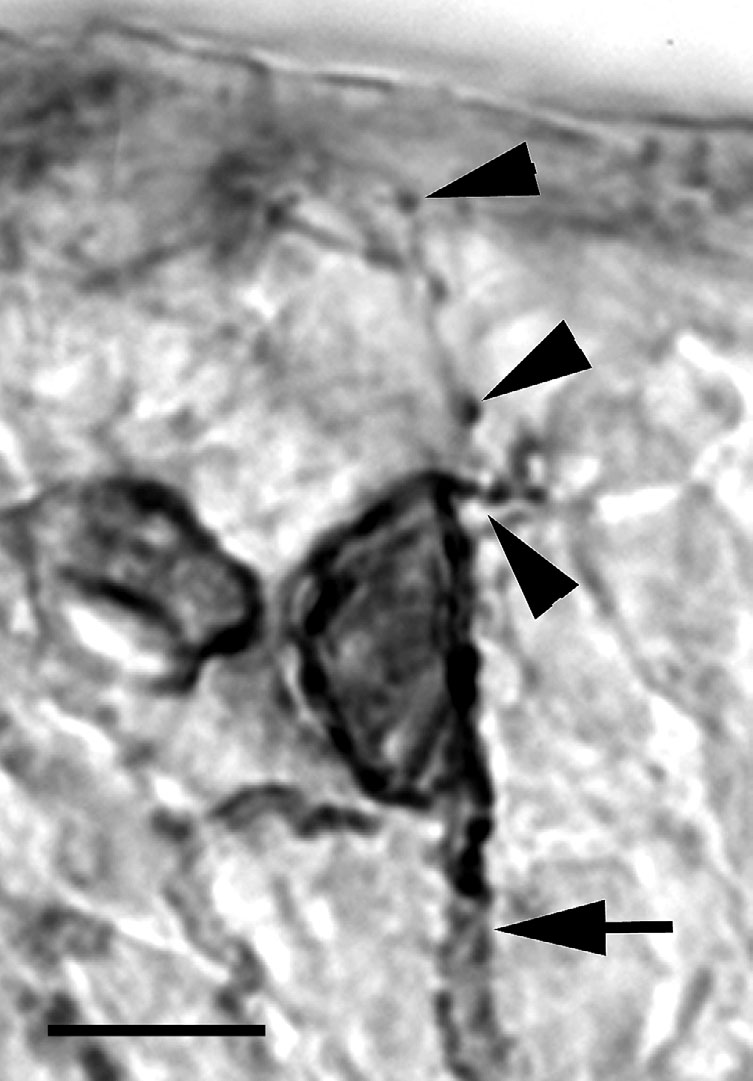
**High magnification micrograph of an agmatine-labeled vomeronasal neuron stimulated with PRF. **Arrowheads indicate punctate labeling in the axon of this neuron, which is projecting toward the lamina propria of the vomeronasal mucosa. The dendrite (arrow) is much thicker than the axon. Bar = 10 μm.

**Figure 4:**
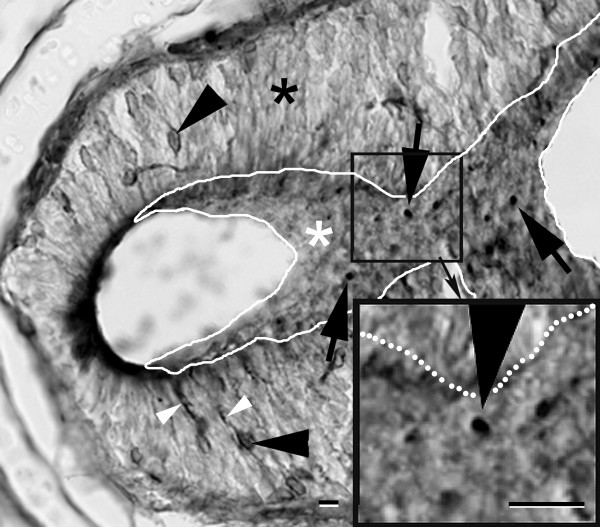
**Low magnification micrograph of female *P. shermani *vomeronasal mucosa showing the area where the epithelium makes a ninety degree turn, allowing for a tangential view of the epithelial surface (area within white line; white asterisk). **This specimen was exposed to PRF. Agmatine-labeled cell bodies (black arrowheads) can be seen in the cross-sectional region of the epithelium (black asterisk). The dendrites (small white arrowheads) of these cells project to the epithelial surface where the dendritic knob appears as a black dot on the epithelial surface (black arrows). The INSET shows a magnified view of the boxed region of epithelial surface with several labeled dendritic knobs. Bars = 10 μm.

Application of PRF to female *P. shermani *salamander snouts resulted in fairly intense labeling of a large population of vomeronasal neurons (Fig. 1A). Labeled neurons were dispersed throughout the vomeronasal epithelium from rostral to caudal levels of the organ and were observed at superficial to deep laminae of the vomeronasal epithelium. Application of control saline produced faint labeling of a few vomeronasal neurons (Fig. 1B). Agmatine labeling was seen in the dendrite, cell body and axon of the vomeronasal receptor cells (Figures 2 and 3), and frequently appeared as round vesicular structures within the dendrite (Figure 2, arrowheads) and axon (Figure 3, arrowheads). Cell bodies had an average diameter of approximately 10 μm. Labeled dendritic knobs were observed at the surface of the epithelium (Figure 2, arrow; Figure 4); these knobs had an average diameter of 2.5 μm. No significant labelling was seen in the main portion of the nasal cavity which is lined with olfactory epithelium. This result was expected since delivery of fluid to the nasolabial groove directs chemosensory stimuli into the vomeronasal organ.

### *Experiment 2. PMF application *– Histological observations

**Figure 5:**
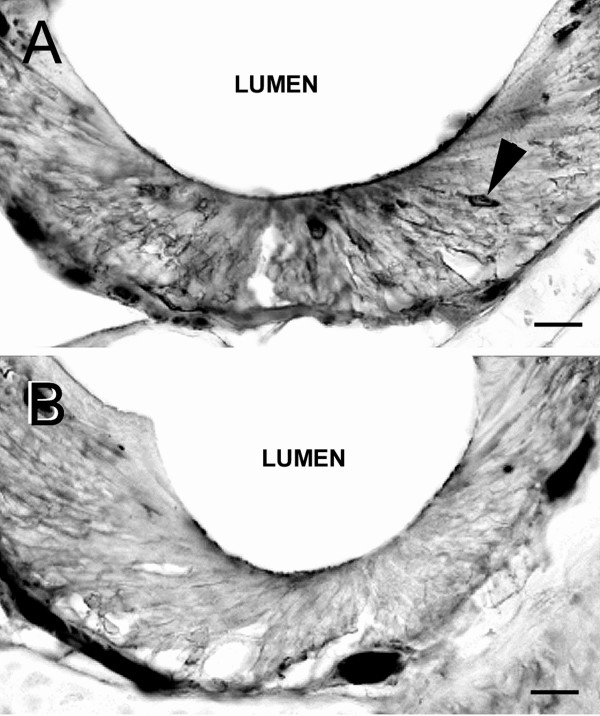
Micrographs of immunocytochemically labeled coronal sections of female *P. shermani *vomeronasal epithelium (VNE) demonstrating agmatine uptake by stimulation with PMF. (A) or a saline control solution (B). (A) Application of PMF resulted in the labeling of a moderate number of vomeronasal receptor neurons (arrow head) throughout all depths of the epithelial layer. Labeling of vomeronasal neurons by PMF was less intense than that by PRF. (B) Application of saline produced very few lightly labeled vomeronasal receptor neurons. Bars = 40 μm.

**Figure 6:**
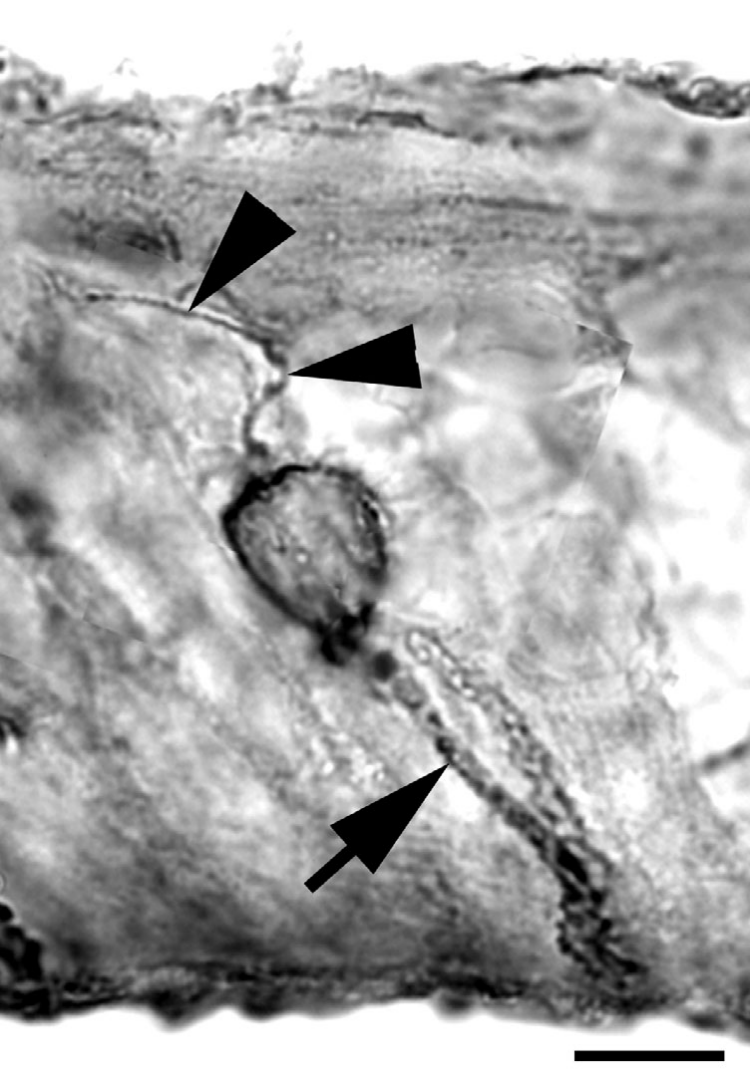
**High magnification micrograph of an agmatine-labeled vomeronasal neuron stimulated with PMF. **The entire cell body, dendrite (arrow) and axon (arrowheads) are labeled. Bar = 10 μm.

Application of PMF to female *P. shermani *also produced labeling of a population of vomeronasal neurons with a distribution similar to those stimulated by PRF (Figure 5A). However, the labeling did not seem to be as intense as with exposure to PRF. Exposure to the control saline solution produced very faint labeling of a few cells (Figure 5B). As with PRF, agmatine label was observed in the cell body, dendrite and axon (Figure 6).

### *Experiment 1 and 2. PRF and PMF *– Statistical Observations

**Figure 8:**
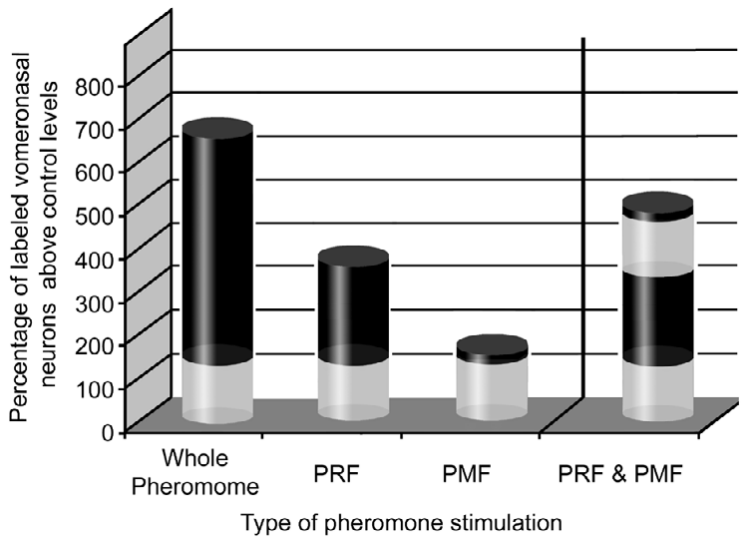
**Graphical representation of the percent number of labeled vomeronasal neurons above control levels for animals exposed to whole pheromone (data from Wirsig-Wiechmann et al., 2002), or to the protein components PRF and PMF. **Control levels of labeling are set at 100% (grey portion of the cylinder) and pheromone stimulation levels (illustrated as the black portion of the cylinder) include the control level. The percentage of cells labeled with the combined stimulation of PRF & PMF is shown as the stacking of the individual cylinders for PRF and PMF, and illustrates the proximity of percent labeling compared to that of the whole pheromone.

Both PRF and PMF stimulated a greater number of neurons than did saline (Figure 8). The number of labeled vomeronasal neurons was significantly greater in the five female salamanders exposed to PRF (mean = 327 neurons, SD = 97.7) than in females exposed to the control saline solution (mean = 76 neurons, SD = 14.1; Figure 3; *t *= 5.68, df = 8, *P *= 0.0002). Likewise, the number of labeled vomeronasal neurons was greater in the five female salamanders exposed to PMF (mean = 222 neurons, SD = 110.8) than in females exposed to saline (mean = 99 neurons, SD = 54.2; Figure 5; t = 2.24, df = 8, p = 0.03). In relation to the total number of neurons in the vomeronasal organ, PRF activated approximately 2% of the neurons, while PMF activated approximately 1% of the neurons.

### Comparison of the data from PRF-stimulated, PMF-stimulated and whole pheromone-stimulated animals

Labeled cells in the saline control group represent cells that are either spontaneously activeor that may respond to agmatine as an odorant. We have assumed that the numbers of cells responding in this spontaneous fashion would remain relatively constant between experiments and that any change in the relative number of these neurons would be a function of the labeling procedure. Based on this assumption, our experimental data (number of neurons labeled following exposure to a chemosensory stimulant) can be standardized by expressing this number as a percentage of control data, and inter-experimental comparisons can then be made. This standardization procedure is necessary since our individual experiments are conducted independently from one another.

The mean experimental data (mean number of labeled vomeronasal neurons) from Experiments 1 (PRF) and 2 (PMF) of this study, and from our previously published study using whole pheromone [24] were expressed as a percentage of control group mean data. When expressed as a percentage of control numbers, whole pheromone (containing both PRF and PMF) produced labeling 618% above control levels while PRF produced 330% labeling and PMF produced 124% labeling above the respective control group levels. The percentage values from PRF and PMF groups combined was 454% labeling above control values.

## Discussion

Agmatine uptake into neurons is a method for visualizing neural stimulation accompanied by the opening of cation channels [25,26]. In this study we used agmatine uptake to identify chemosensory neurons that are activated by each of the two major protein components of male *P. shermani *pheromone: PRF and PMF, so named because of their different effects on female salamander behavior. Vomeronasal organ labeling in this study demonstrated similar characteristics to that of our previous study using the whole pheromone as the stimulus. Labeled vomeronasal neurons appear in all laminae of the sensory epithelium, are observed throughout the entire organ and show dark to light labeling of the cytoplasm in the dendrite, cell body and proximal axon. Visualization of the surface of the vomeronasal epithelium also has shown that individual dendritic knobs contain agmatine, illustrating that the uptake is highly specific. The labeling intensity of vomeronasal neurons was generally greater for PRF than for PMF.

Previous research on a variety of vertebrate species has demonstrated heterogeneity of cell types in the vomeronasal organ based on their molecular characteristics [21]. Vomeronasal receptor neurons express receptors from two main multigene families, V1R and V2R receptors, [27-30]. Vomeronasal receptor neurons also express different G-proteins, Giα2 and Goα, each of which are confined to the superficial and deep layers of the vomeronasal epithelium respectively, and appear to be co-expressed with the V1R and V2R family of receptors, respectively [31-34]. Finally, there is evidence that the vomeronasal neurons use an effector system that is different than that of the olfactory system (IP_3_, [35-38]). This heterogeneity of receptor cell characteristics may reflect segregation of response characteristics into several broad categories, the exact nature of which is not completely clear to date. The cells that respond to PRF and PMF appear to be evenly distributed in the vomeronasal epithelium. Therefore, these cells could not be classified as belonging to V1R and V2R cell groups, if *P. shermani *does have these cell groups.

Of the multiple protein components that comprise the male *P. shermani *whole pheromone, the two main proteins, PRF and PMF, account for approximately 85% of the proteins found in whole pheromone [19]. The presence and relative proportion of these two proteins has been highly consistent over multiple years of obtaining gland extracts from *P. shermani *salamanders (Richard C. Feldhoff, unpublished observations). If we consider that the whole pheromone should stimulate 100% of pheromone-responsive vomeronasal neurons, then we could expect PRF and PMF together to stimulate about 85% of these neurons. Using the data from the whole pheromone study (618% labeling above background) and the data from the two present studies, we find that PRF and PMF together (330% + 124% = 454% labeling above background) produce neural labeling that is 73.5% of that produced by whole pheromone (454% **/ **618% = 73.5%). This supports our hypothesis that each protein component independently binds to a specific type of receptor and activates a separate population of female *P. shermani *vomeronasal neurons. In addition, due to small size of these pheromones they may be able to access other parts of the nasal cavity besides vomeronasal areas. The vomeronasal organ could be the initial or principal area of stimulation, but not the only site of action.

Plethodon Receptivity Factor is synthesized in the mental glands of male plethodontid salamanders that deliver pheromone by direct contact between the male's gland and the female's nares. PRF also is found in mental gland secretions in Plethodon species (such as *P. cinereus*) that deliver pheromone by swabbing mental gland secretions on areas of the female's dorsum that have been abraded by the male's premaxillary teeth [39], thus "injecting" the molecule systemically. PRF exhibits sequence homology to the IL-6 cytokines, most notably to neurotropin, and displays the characteristic four-α-helix bundle in the protein [40]. This structural and sequence homology suggests that this biomolecule originally evolved as a cytokine and perhaps is used as such systemically in the salamander species that scratch. In *P. sherman*i, the PRF may have taken on an additional chemosensory role in courtship behavior [41]. This molecular relationship, a molecule designed as a cytokine and pheromone, would represent a novel finding in vertebrates. However, such a dual role has been described for the bacterium, *Micrococcus luteus*, which synthesizes a peptide that functions as a cytokine and a pheromone [42-45]. In addition, the ciliated protozoan, *Euplotes raikovi*, secretes a "pheromone" that binds to cytokine receptors [46]. It is still not clear whether PRF acts centrally in *P. shermani*. It is highly probable that the behavioral effects of exogenously applied PRF in female *P. shermani *are mediated through the chemosensory pathways in the brain [18]. However, whether cytokines can produce direct or indirect behavioral effects has yet to be tested.

The exact function of PMF is not yet known, although in behavioral experiments females take longer to mate when they receive exogenously applied PMF. The structure of PMF is similar to the toxin, alpha bungaro-toxin, a compound found in snake venom that binds tightly to nicotinic receptors and causes paralysis of skeletal muscle. Because PRF and PMF in combination act to reduce mating time (increase receptivity of the female) one possible function of PMF would be to relax the female. This relaxing effect could serve to reduce the risk that other stimuli would distract the female from courtship. Thus, the two protein components of the pheromone solution may produce both sedative (PMF) as well as stimulatory (PRF) effects that synergistically facilitate courtship. Other animals have been shown to produce pheromones that also act as paralyzing agents. Two *Metapone *ant species from Madagascar produce a trail pheromone that is synthesized in a poison gland and that they also use as a paralyzing agent for capturing prey [47]. The poison gland of another ant species, Harpagoxenus sublaevis, synthesizes sex pheromones [48]. Further studies are needed in Plethodon salamanders to determine whether pheromone components delivered during courtship have physiological actions beyond the vomeronasal organ.

## Conclusion

In summary, we have used the agmatine uptake method to show that the two protein components in mental glands of male *P. shermani *each stimulate a set of vomeronasal receptor neurons. This VNO stimulation suggests that information from each component is processed by the accessory olfactory bulb and contributes to the behavioral effects on female salamander courtship behavior. Further studies are needed to ascertain whether these two protein components also enter the general circulation to influence peripheral physiological responses to the male.

## Methods

### Animals

Ten female salamanders (*P. shermani*) were used as olfactory subjects in each of the two experiments to test the effects of PRF and PMF. Animals were collected from Wayah Bald (Macon County, NC) during August, the beginning of the plethodontid mating season. Animals were maintained individually, each in a plastic box (31 × 17 × 9 cm) lined with moist paper towels and containing crumpled moist towels as refugia. The salamanders were exposed to a 14:10 light/dark illumination schedule and were fed wax worm larvae or fruit flies.

### Isolation of male pheromone

Isolation of the protein components of the male pheromone (cf [19]) was conducted to test the effects of each protein component on vomeronasal receptor neurons (Wirsig-Wiechmann, et. al. 2002). Mental glands were removed from approximately 120 male salamanders following anesthesia in a mixture of 7% ether in water. Glands were placed in a solution of acetylcholine chloride (AChCl) for approx. 60 min. The extracted solution was then centrifuged for 10 min (at 14,000 × g), the supernatant was collected and centrifuged again for 10 min. Supernatant was frozen at -80°C until used. Gland extract was further processed to obtain purified 7 kDa and 22 kDa proteins. Gland extracts were filtered (0.2 μm non-protein binding filter), then applied to a Mono-Q column (FPLC HR 5/5; Pharmacia, Piscataway, NJ) at 50 mM Tris-HCl, pH 8.0. The column was eluted (same buffer) at 1 ml/min using a NaCl gradient (5.0 mM NaCl/min). Enriched pheromone fractions were further purified by re-chromatography on the Mono Q column followed by gel filtration chromatography on a G75 Superfine column (1.6 × 15.5 cm; Pharmacia) previously equilibrated with 0.5× PBS. The protein content of the solution was standardized to 0.7 5 mg/mL (PRF) or 0.5 mg/mL (PMF) in 0.5× PBS so that protein concentration was consistent for all pheromone trials and reflected the relative concentrations of PRF and PMF in the whole pheromone mixture. The purified solutions of 7 kDa and 22 kDa proteins were frozen in aliquots and were thawed just before use.

### Pheromone application to females

#### Experiment 1: PRF application to females

Female salamanders were exposed to solutions containing 3 mM agmatine in 0.9% NaCl (saline control group) or 0.35 mg/ml Plethodon Receptivity Factor in 3 mM agmatine/0.9% NaCl (PRF group). Females from each group (PRF, n = 5; saline, n = 5) were placed in separate clean and dry plastic containers. Two microliters of solution were applied to the female's snout using a P-10 Gilson Pipetman approximately every 2 min over a 45 min period (20 stimulus applications per female). Following PRF or saline applications, 5 microliters of PBS were applied three times over the course of 5 min to female nares to wash away excess agmatine.

#### Experiment 2: PMF application to females

Plethodon Modulating Factor (PMF) was used as the stimulus for this second experiment and all procedures were conducted in the same manner as in experiment 1. Female salamanders were exposed to 3 mM agmatine in 0.9% NaCl (saline control group; n = 5) or 0.2535 mg/ml Plethodon Modulating Factor in 3 mM agmatine/0.9% NaCl (PMF group; n = 5).

### Tissue preparation and immunocytochemistry

Following exposure to pheromone stimuli or saline, female salamanders were killed by decapitation. Heads, with jaws removed, were immersion-fixed overnight in 4% paraformaldehyde-2.5% glutaraldehyde in PBS, pH 7.4. Tissue was then decalcified in DeCal (Decal Corporation, Congers, NY) for three days and cryoprotected in 30 % sucrose in PBS for two days. Pairs of heads (one from the pheromone group and one from the saline group) were frozen in M-1 matrix (Shandon, Pittsburgh, PA), and stored at -80°C until sectioning. Tissue was sectioned (20 μm) in the coronal plane on a cryostat microtome. Five sets of sections were collected so that each section in a set was separated by 100 μm. Sections were stored at -80°C until labeling procedures could be conducted on all tissue in the experiment at the same time. Plastic slide mailers were used for tissue incubations.

Immunocytochemical procedures for labeling agmatine were conducted as previously reported [24]. Briefly, tissues were rinsed in PBS to remove the fixative, incubated for 30 min in preincubation buffer and incubated in rabbit anti-agmatine antisera (1:4000; Chemicon International, Inc., Temecula CA; Lot # 18112624) for three days. One set of sections was labeled with diaminobenzidine (DAB) and another set was labeled with goat anti-rabbit Alexa Fluor 488 antiserum (Molecular Probes, Eugene, OR) and counter-labeled with Hoechst as previously described.

### Histological and statistical analysis

Coronal sections of female salamander head were examined for identifying chemosensory cells containing DAB or fluorescent label. Digital images of chemosensory mucosae were obtained using an Olympus microscope with a SPOT camera (Diagnostic Instruments, Sterling Heights, MI). DAB-labeled vomeronasal neurons were counted in every fifth 20 μm section of salamander head throughout the entire vomeronasal organ, and the total number of labeled neurons in each animal was used for statistical analyses [24]. Neurons were considered labelled if the cytoplasmic labelling intensity in the cell body was visibly higher than background as observed with a 20 × objective. An unpaired Student's *t*-test was used in each experiment to compare the total number of labeled vomeronasal neurons between experimental and control groups (PRF vs. saline data for Experiment 1 and PMF vs. saline data for Experiment 2). Statistical analyses were carried out using online statistical programs [49].

## Authors' contributions

CRWW designed the experiments, prepared the agmatine-stimulant solutions, applied the solutions to the salamanders, killed the salamanders, prepared the tissue for histological sectioning and wrote the bulk of the manuscript including the abstract, background, results and discussion. LDH oversaw the collection of the salamanders and screened the animals for courtship behavior. JMW sectioned the tissue, performed the immunocytochemical procedures, participated in collecting and quantifying the data and wrote some sections of the manuscript. PWF and RCF conducted the biochemical isolation and purification of the pheromone protein components. LDH, PWF and RCF made revisions to the final manuscript.

## Figures and Tables

**Figure 7:**
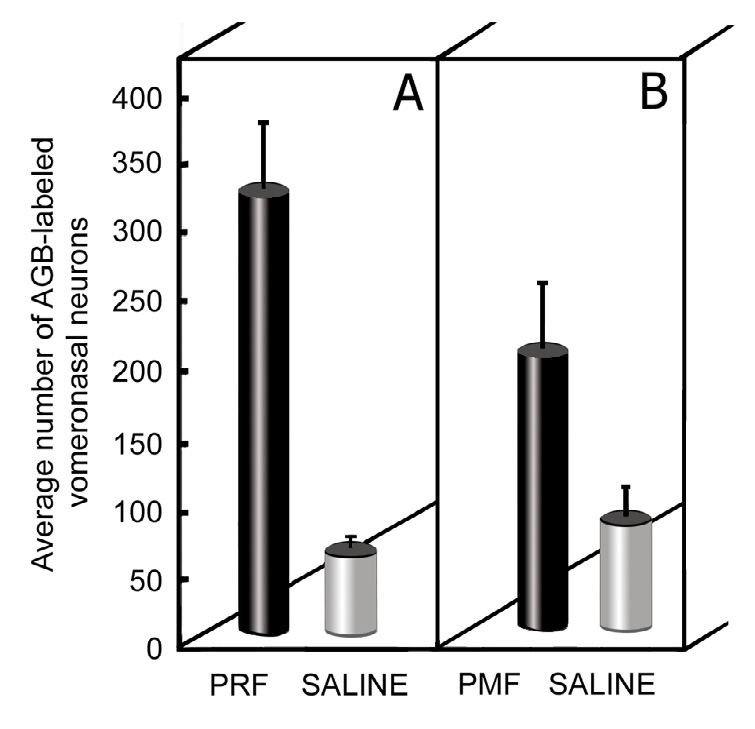
**Graphical representation of the average number of agmatine-labeled vomeronasal neurons that were labeled following stimulation with: (A) PRF or its control saline, and (B) PMF and its control saline. **Both PRF and PMF stimulated significantly more vomeronasal neurons than did saline. Each cylinder represents the average (± SD) number of labeled vomeronasal neurons from five female salamanders.
